# Numerical Simulation of Airflow Fields in Two Typical Nasal Structures of Empty Nose Syndrome: A Computational Fluid Dynamics Study

**DOI:** 10.1371/journal.pone.0084243

**Published:** 2013-12-18

**Authors:** Meng-Yang Di, Zhe Jiang, Zhi-Qiang Gao, Zhi Li, Yi-Ran An, Wei Lv

**Affiliations:** 1 Department of Otolaryngology, Peking Union Medical College Hospital, Peking Union Medical College and Chinese Academy of Medical Sciences, Beijing, China; 2 Department of Mechanics and Engineering Science, College of Engineering, Peking University, Beijing, China; University of Arizona, United States of America

## Abstract

**Background:**

The pathogenesis of empty nose syndrome (ENS) has not been elucidated so far. Though postulated, there remains a lack of experimental evidence about the roles of nasal aerodynamics on the development of ENS.

**Objective:**

To investigate the nasal aerodynamic features of ENS andto explore the role of aerodynamic changes on the pathogenesis of ENS.

**Methods:**

Seven sinonasal models were numerically constructed, based on the high resolution computed tomography images of seven healthy male adults. Bilateral radical inferior/middle turbinectomy were numerically performed to mimic the typical nasal structures of ENS-inferior turbinate (ENS-IT) and ENS-middle turbinate (ENS-MT). A steady laminar model was applied in calculation. Velocity, pressure, streamlines, air flux and wall shear stress were numerically investigated. Each parameter of normal structures was compared with those of the corresponding pathological models of ENS-IT and ENS-MT, respectively.

**Results:**

ENS-MT: Streamlines, air flux distribution, and wall shear stress distribution were generally similar to those of the normal structures; nasal resistances decreased. Velocities decreased locally, while increased around the sphenopalatine ganglion by 0.20±0.17m/s and 0.22±0.10m/s during inspiration and expiration, respectively. ENS-IT: Streamlines were less organized with new vortexes shown near the bottom wall. The airflow rates passing through the nasal olfactory area decreased by 26.27%±8.68% and 13.18%±7.59% during inspiration and expiration, respectively. Wall shear stresses, nasal resistances and local velocities all decreased.

**Conclusion:**

Our CFD simulation study suggests that the changes in nasal aerodynamics may play an essential role in the pathogenesis of ENS. An increased velocity around the sphenopalatine ganglion in the ENS-MT models could be responsible for headache in patients with ENS-MT. However, these results need to be validated in further studies with a larger sample size and more complicated calculating models.

## Introduction

Empty Nose Syndrome (ENS) was initially proposed by Eugene Kern and Monika Stenkvist in 1994, to name a group of syndromes relevant to turbinate injuries or losses[1,2]. According to the Anglo-American usage[3], there are three subtypes of ENS: ENS-inferior turbinate (ENS-IT), ENS-middle turbinate (ENS-MT) and ENS-both, due to the pathological changes of the inferior, middle, and both turbinates, respectively. Typical manifestations differ among subtypes. Paradoxical nasal obstruction, dryness and crusting are the leading symptoms in patients with ENS-IT or ENS-both[1], while pain associated with breathing is predominant among those with ENS-MT[4]. The different manifestations label ENS-MT as a controversial subtype[1-4].

The pathogenesis of ENS has not been elucidated yet[1-4]. The changes of nasal aerodynamic features are postulated to be responsible for the development of syndromes of ENS[1]. However, till now there is a lack of evidence to support the hypothesis. As far as we know, only three computational fluid dynamic (CFD) studies[5-7] have been conducted to explore the changes of aerodynamic features in the postoperative nasal structures of inferior turbinectomy- the typical pathological structure of ENS-IT. The changes reported in these studies were not all consistent with each other. For instance, the velocities and the wall shear stresses widely decreased with inferior turbinectomy in two of the studies[5,6], while increased in the third study[7]. Additionally, since those previous studies only included one or two subjects, the extrapolation of the results would be greatly impaired by the potentially great nasal anatomical variations among individual people. As mentioned previously, patients with ENS-MT usually present atypical symptoms, compared to those with ENS-IT or ENS-both, which makes the investigation on the nasal aerodynamic changes in ENS-MT especially compelling and meaningful. However, few such studies have been carried out so far in the typical nasal structures of middle turbinectomy, leaving a knowledge gap on the nasal airflow features in ENS-MT[1]. 

Further evidence on the aerodynamic features in the nasal structures of ENS patients is in great need to help doctors to get a better understanding of the pathogenesis of ENS and to make proper therapeutic plans for the patients. Therefore, this study aims to investigate the common airflow characteristics in the typical nasal structures of ENS-IT and ENS-MT in 7 different individuals.

## Methods

### Ethics statement

The protocol of the study has been approved by the ethical committee of Peking Union Medical College Hospital. Each subject signed informed consent before recruited in the study.

### Study subjects

Seven healthy male adults (age range: 29-41) without history of chronic nasal or sinus diseases (atrophic rhinitis, nasal septum deviation, turbinate hypotrophy, etc.) were included in this study and were sequentially numbered as 1-7. None of them had experienced any acute upper respiratory infections three months before the study. All the 7 subjects were scored 0 in both the Visual Analogue Scale (VAS) and the Sino-nasal Outcome Test-20 (SNOT-20). Nasal structures and mucosa were normal in nasal inspection in all the subjects.

### Normal nasal structures

The nasal cavity geometry was obtained through a computed tomography (CT) scan. High-resolution CT (Siemens, German) of sino-nasal areas was performed in each of the 7 subjects. The layer interval of the CT scan was 0.6 mm. CT scan for each subject was finished within 30 minutes, to reduce the influence of nasal period on the shape of the turbinates. The boundaries of the nasal cavities and all the sinuses were numerically extracted. Smoothing of the extracted surfaces was performed to facilitate mesh generation of the three-dimensional models and reduction of computational effort as well as the increase of computational efficiency. 

### ENS nasal structures

The nasal structures of bilateral radical inferior turbinectomy and bilateral radical middle turbinectomy were chosen to mimic ENS-IT and ENS-MT, respectively. In the latter structures, the horizontal parts of the middle turbinates were removed, while the vertical parts were preserved. All the virtual operations were performed by one otorhinolaryngologist by following the standardized procedures, to ensure the relative consistency among different models. 

The unstructured tetrahedral cells were generated by the mesh generator, ICEM CFD (ANSYS, Inc.). In consideration of the complicated structures in the nasal cavity and relatively low airspeeds corresponding to quiescent respiratory airflow, prism layers were not created near the walls, which might lead to a poor mesh quality and increase the time and memory consumption. The ENS-IT model during inspiration of No. 3 was taken as a representative for the convergence test. Analysis of grid convergence was performed by comparing the velocity profiles for an arbitrary line in the geometry for a constant inhalation flow rate of 15 L/min, for each of four different grid sizes (approximately 435 000, 1 100 000, 2 000 000 and 3 000 000 tetrahedral elements). Similar profiles were obtained for the two higher grid sizes. The convergence trend was clear and the 2 000 000 element grid was thought to be sufficient to resolve the relative change tendency in flow fields in the nasal cavity. The total number of cells, ranging from 1 619 000 to 2 066 000 for different cases in our study, was at the same grid level. 

### Numerical simulation

The aerodynamic parameters (pressure, velocity, streamlines and wall shear stress) were obtained by solving Navier-Stokes equations and continuity equations, using a commercial CFD code of FLUENT. The second-order upwind scheme was used in spatial discretization. The pressure-velocity coupling was resolved through the SIMPLE method. In all the calculations carried out, the airflows were assumed to be incompressible and steady. The inlet plane was extracted below the nasopharynx, and the outlet plane close to the nostril. A uniform velocity normal to the inlet plane was specified by the quiescent cyclic respiratory airflow rate of 15 L/min[6,7] through the entire nasal cavity. The inlet velocity was of negative value during the period of inspiration, and positive during expiration. The gauge pressure at the outlet was set equal to one atmosphere pressure. The sino-nasal wall boundary condition was assumed as rigid and no-slip[6]. The Reynolds number based on the inlet velocity and the nasopharynx diameter was approximately 1300, indicating that the flow was predominantly laminar. To make it reasonably simple, the laminar model was used in the simulation.

Most of the solutions were well-converged after 2000 iterations with 8 CPUs. All residuals were below 1e-06 and the surface monitor of mass flow rate at the inlet almost unchanged. 

**Figure 1:**
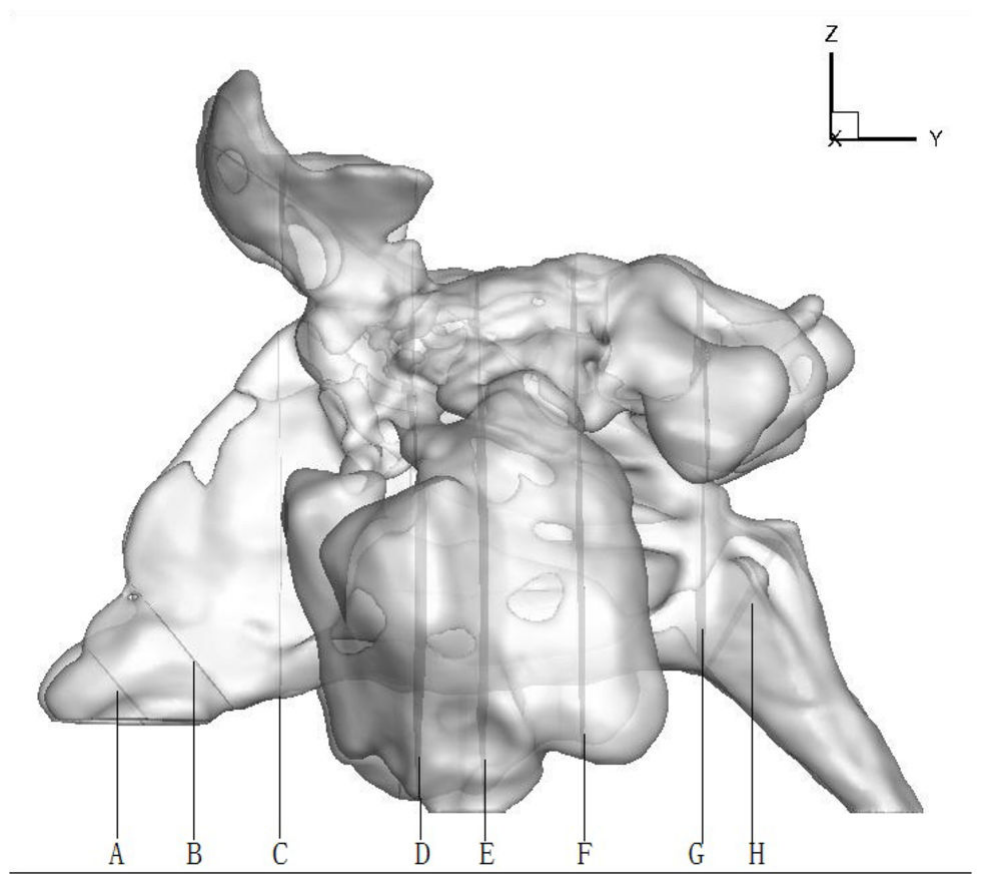
Representative planes in the noses. A: Nasal vestibule, B: Nasal valve, C: Head of the inferior turbinate, D: Head of the middle turbinate, E: Middle of the inferior turbinate, F: Posterior portion of the inferior turbinate, G: choanal, H: Nasopharynx. The model in the figure is of the No. 3 subject.

Eight cross sections (Figure 1) were extracted in the 21 models. All the cross sections were approximately perpendicular to the local airflow directions. The aerodynamic parameters of the 8 planes were calculated and compared between the normal and the pathological structures. 

In the initially obtained aerodynamic features, the maximum velocities noticeably increased within the choanal planes in the ENS-MT nasal structures (but not in the ENS-IT structures, see details in the result part), which approached the sphenopalatine ganglion- the suspected origin of headache in ENS-MT[1]. In order to get the maximum velocities around the sphenopalatine ganglion more accurately, the space around pteryopalatine fossa was extracted, surrounded by the lateral nasal wall, lower portion of the anterior wall of sphenoid sinus, and the back parts of the (previous) middle and superior turbinates and the (previous) middle meatus. The inner sidewall of the extracted space was 5.51±0.95mm apart from the nasal septum on average in the 7 subjects. A relatively broader scope was used in our study, as there are potential anatomical variations in the location of sphenopalatine ganglion in different individuals and the stimulations to both the ganglion and its accessory nerves would lead to headache. Maximum velocities were measured by the aforementioned method in that area. 

A decreased airflow rate was qualitatively observed in the middle and superior meatuses as well as the upper portion of the common meatus in the ENS-IT nasal structures (see details in the result part). We extracted a representative coronal plane, plane of F in each model and calculated the airflow rates in the abovementioned area.

## Results

The aerodynamic parameters were illustrated as follows: 1. Total pressure difference and nasal resistance (the latter one is calculated as the total pressure difference divided by the flow rate); 2. Velocity; 3. Streamlines and air flux; 4. Wall shear stress distribution, in each of the 8 planes in the 21 models (7 normal structures, 7 ENS-IT, and 7 ENS-MT). The parameters of the pathological structures of ENS-MT and ENS-IT were compared respectively with the corresponding normal ones. 

The inspiratory phase is more essential for the nasal respiratory function. Additionally, the changes of the aforementioned parameters were similar in the two phases in our study. Thus, we mainly illustrated the changes in the inspiratory phase in this part.

The differences in the values of the parameters (nasal resistance, maximum velocity and airflow rate) between groups (normal and ENS-MT/ENS-IT) approximately satisfied the normal distribution. Thus, paired t-test (two tailed) was applied to calculate the t and *P* values by SPSS 17.0 software (IBM, USA). *P*<0.05 was defined as statistical significance. 

**Figure 2:**
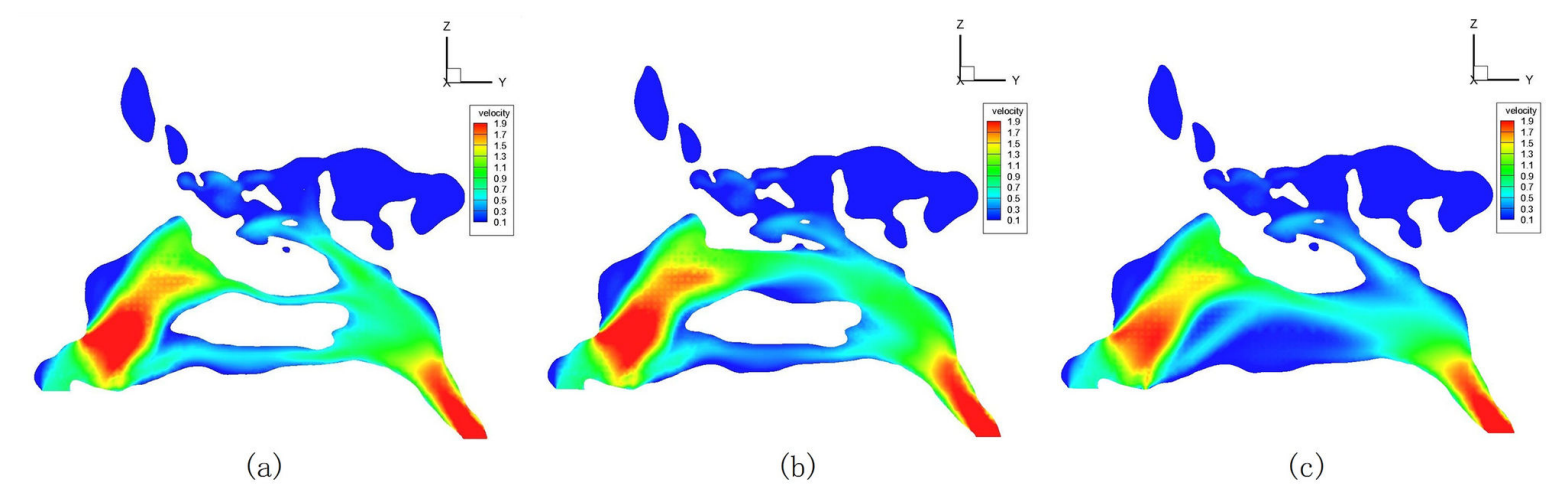
Velocity distribution during inspiration in normal (a), ENS-MT (b) and ENS-IT (c) models (No. 3).

**Figure 3:**
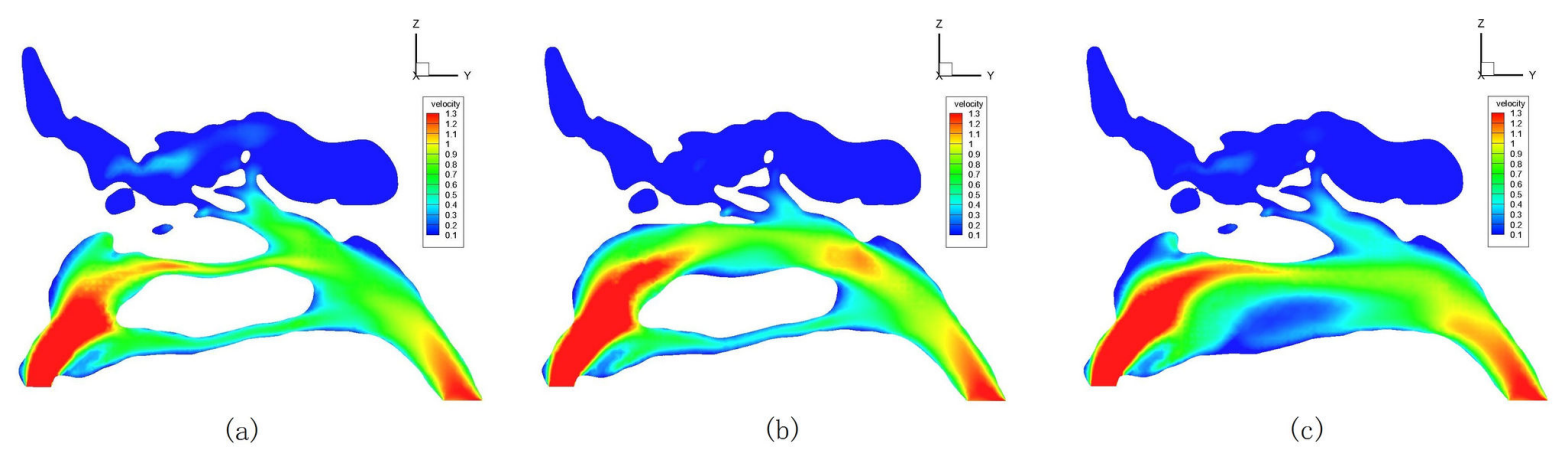
Velocity distribution during inspiration in normal (a), ENS-MT (b) and ENS-IT (c) models (No.2).

**Figure 4:**
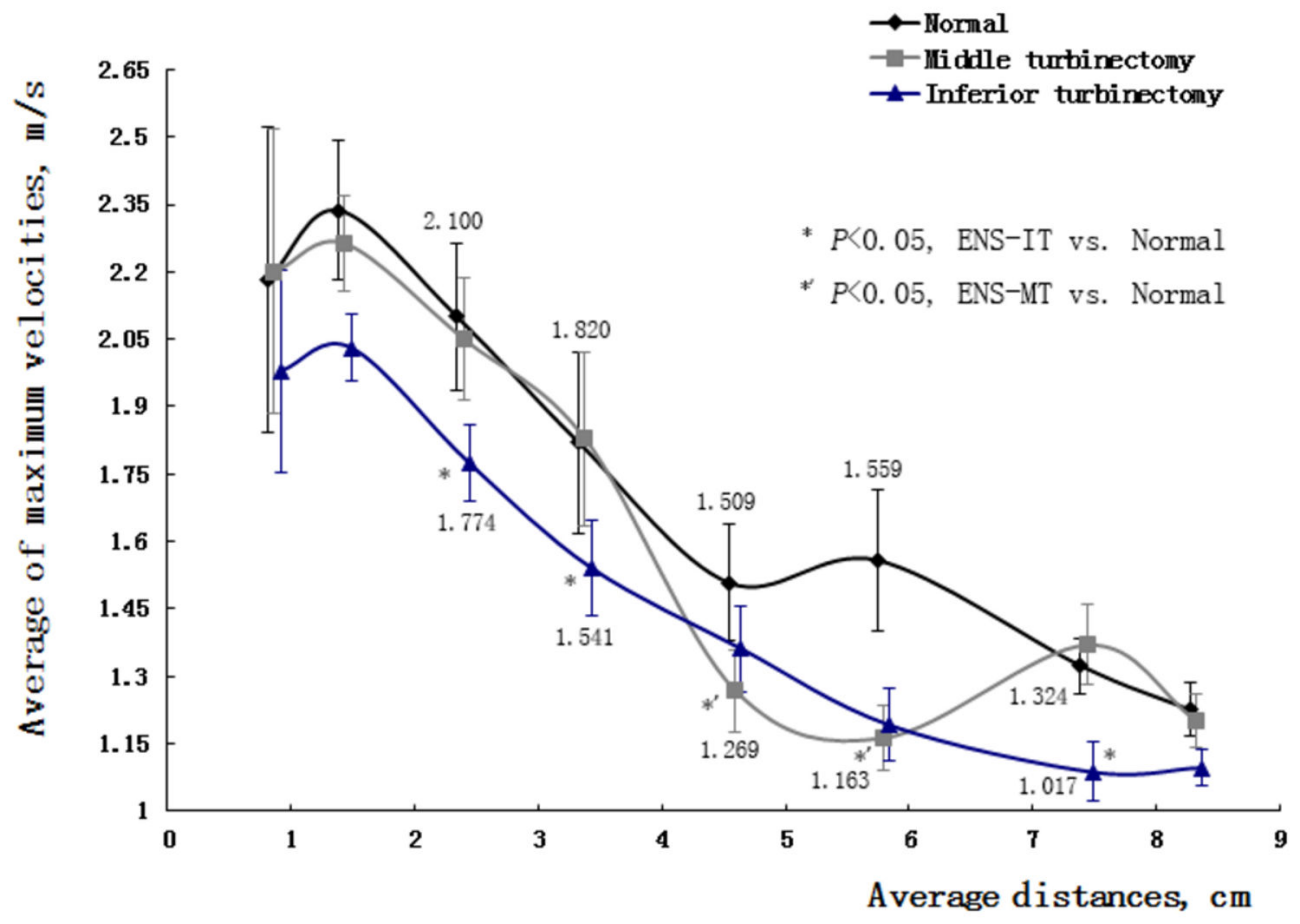
Average of maximum velocities on cross sections in 7 normal, ENS-MT and ENS-IT models during inspiration.

1. Nasal resistances changed proportionally with the total pressure differences in our study, due to the fixed airflow rate. Nasal resistances decreased significantly in both ENS-MT (nasal resistance decrease=0.0067±0.0059Pa·s/cm^-3^) and ENS-IT (nasal resistance decrease=0.0134±0.0100Pa·s/cm^-3^) models, when compared with the corresponding normal structures (Table 1). Nasal resistance of ENS-IT was significantly lower than that of ENS-MT (*P*=0.007).

2. In the ENS-MT models, velocity distributions changed little in the velocity contours, only with relatively low velocities shown in the areas where the middle turbinate previously resided (Figure 2 and 3). Correspondingly, the means of the maximum velocities in the eight typical cross sections, compared with those in normal structures, changed little within a broad area of the nasal valve, head of inferior turbinate, inferior meatus, and the nasopharynx areas. The maximum velocities decreased significantly at the middle-posterior cross sections of the middle turbinate (*P*_ENS-MT_=0.036, 0.008) (Figure 4). 

**Table 1:** Nasal resistances, velocities around sphenopalatine ganglion, and airflow rates through nasal olfactory area in normal, ENS-MT and ENS-IT models.

	Normal	ENS-MT	ENS-IT
	( mean±SD)	( mean±SD)	( mean±SD)
Nasal Resistance^1^, 10^-2^ Pa·s/cm^3^	2.19±1.09	1.51±0.61^*^	0.84±0.29^*^
Nasal Resistance^2^, 10^-2^ Pa·s/cm^3^	2.27±0.90	1.71±0.49^*^	0.99±0.20^*^
Velocities**^*1**†*^** , m/s	1.14±0.27	1.34±0.26^*^	--**^*¶*^**
Velocities**^*2**†*^**, m/s	1.52±0.44	1.74±0.44^*^	--**^*¶*^**
Airflow rates**^*1**§*^, %**	49.76±7.44	63.56±4.66^*^	23.49±11.10^*^
Airflow rates **^*2**§*^**, **%**	52.43±5.61	70.21±6.32^*^	39.24±10.34^*^

^1^ The corresponding values of inspiration;

^2^ The corresponding values of expiration;

^§^ Airflow rates around olfactory nerve endings in the nasal cavities;

^†^ Velocities around the sphenopalatine ganglion;

^*^ Statistically significant in paired-t test (two-tailed, *P*<0.05);

^¶^ Not calculated (for reasons refer to the result part).

In the ENS-IT models, velocities decreased more broadly and more greatly in the velocity contours than ENS-MT, with low velocities shown in the areas where the inferior turbinate previously resided (Figure 2). The maximum velocities decrease significantly on the anterior-middle cross sections of the inferior turbinate and choanal (*P*_ENS-IT_=0.031, 0.021, 0.014) (Figure 4). (The velocity profile of model No. 3 was taken as a representative of the changes of velocity distribution in Figure 2).

**Figure 5:**
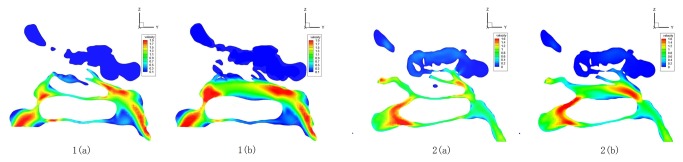
Velocity distributions during expiration in normal and ENS-MT models (No. 1 and 4). 1(a) Normal model of No. 1, 1(b) ENS-MT model of No. 1. 2(a) Normal model of No. 4, 2(b) ENS-MT model of No. 4.

**Figure 6:**
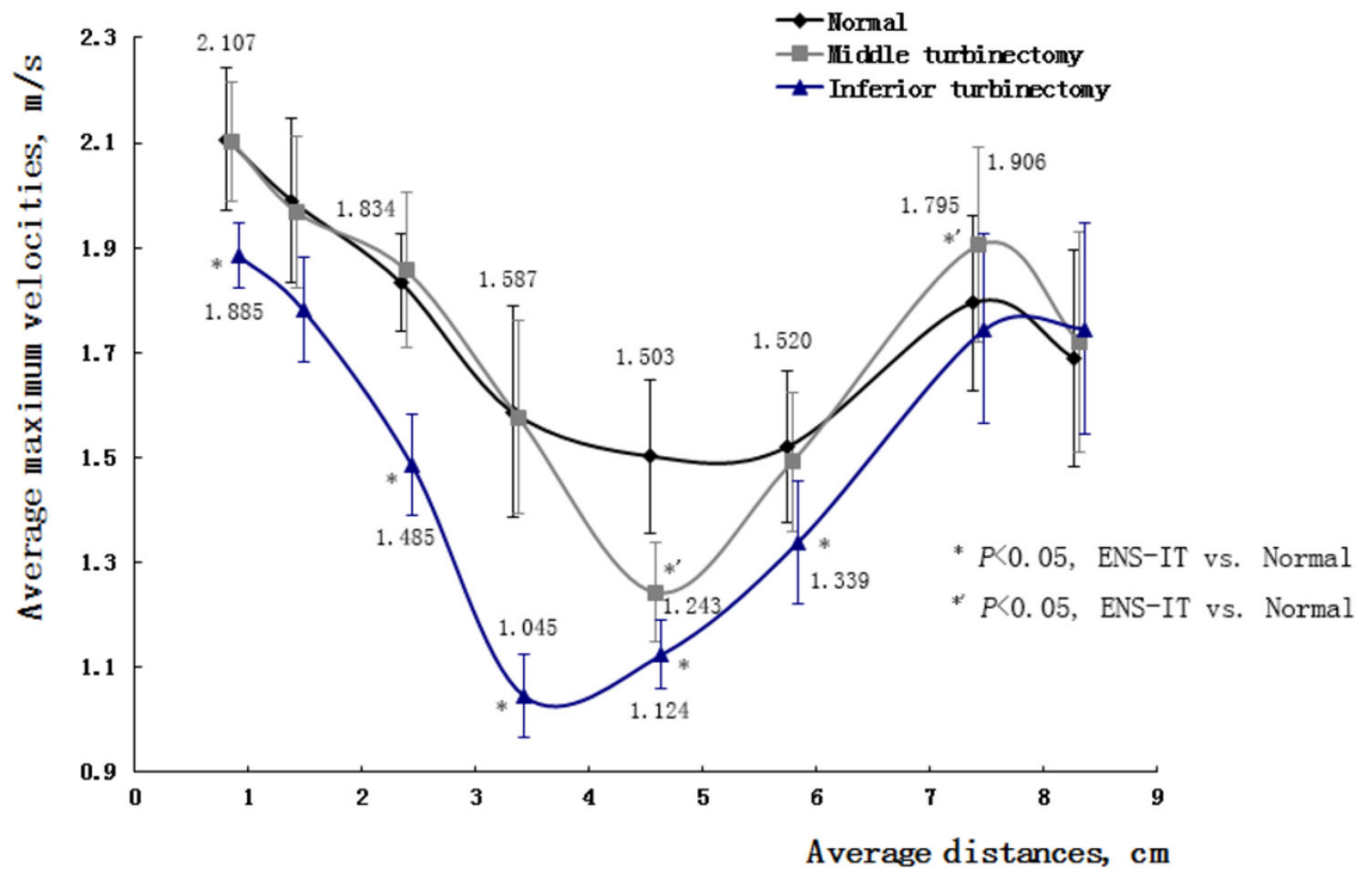
Average of maximum velocities on cross sections in 7 normal, ENS-MT and ENS-IT models during expiration.

Noticeably, velocities increased in the upper-posterior area behind the inferior turbinate in 4 of the ENS-MT models during inspiration, and increased in 6 models during expiration, as shown in the velocity contours (Figure 5). Consistently, the means of the maximal velocities within the choanal planes increased in both phases, but the increase was only significant during expiration (*P*=0.020) (Figure 4 and 6). As the area with increased velocities was close to sphenopalatine ganglion, a reasonable suspect would be an increase in velocities around the ganglion. The suspect was proved in our calculations that the means of the 7 subjects significantly increased during both phases in ENS-MT (velocity increase during inspiration=0.20±0.17m/s, velocity increase during expiration=0.22±0.10m/s) (Table 1). We did not attempt to obtain the velocities in that area in the ENS-IT models, since there was no evidence of increased velocities either qualitatively in the contours or quantitatively in the choanal planes.

**Figure 7:**
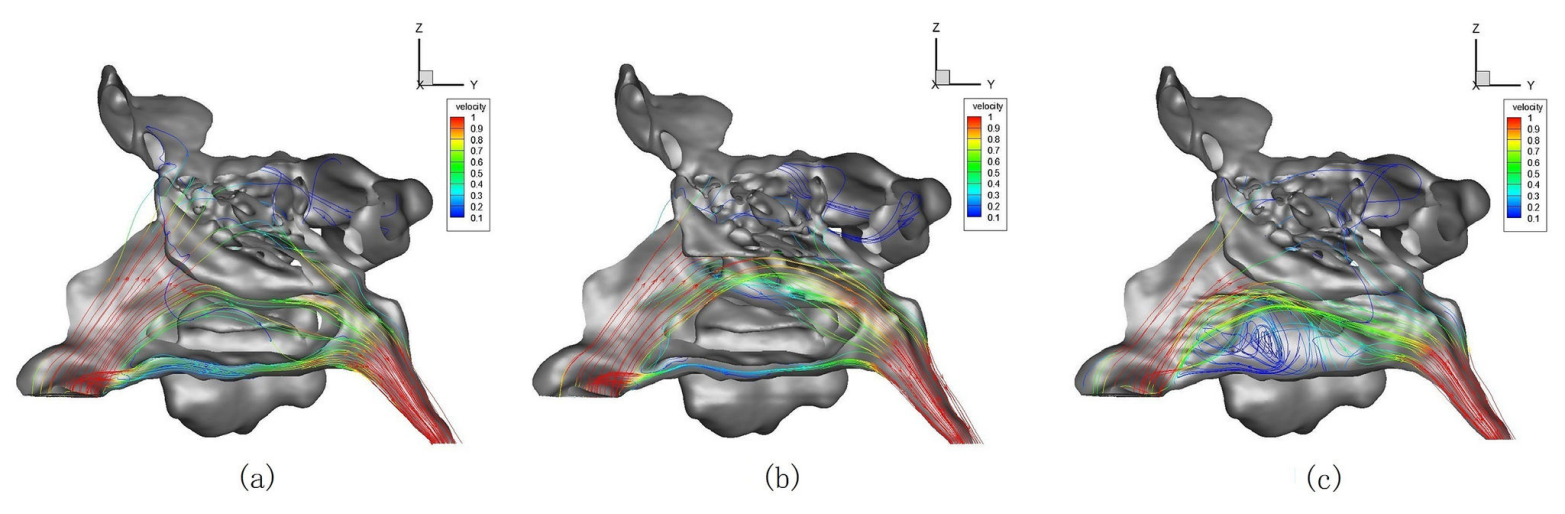
Air fluxes distributions and trajectories in normal (a), ENS-MT (b) and ENS-IT (c) models (No. 3).

3. Changes of the air flux distributions and the streamlines were similar in all the 7 ENS-MT/ENS-IT models. Both the ENS-MT and ENS-IT structures of model No. 3 were representatively illustrated in Figure 7. 

In the normal models, most of the airflow passed through the middle-upper part of the common meatus and the middle meatus. In the ENS-MT models, air flux distributions changed slightly, compared to the normal ones, only with more fluxes shown in the middle-upper portion of where the middle turbinate previously resided. In the ENS-IT models, most airflow passed through the middle-upper portion of where the inferior turbinate previously resided. Air fluxes decreased greatly in the other parts of the nasal cavity. When we calculated the overall airflow rates in both middle and superior meatuses and upper portion of the common meatuses in all the 7 subjects, the rate significantly decreased by 26.27%±8.68% and 13.18%±7.59%, respectively, during inspiration and expiration in the ENS-IT nasal structures (Table 1).

In the normal models, laminar flows were predominant, with vortexes mainly found in the sinuses. In the ENS-MT models, streamlines changed little, while in the ENS-IT models, streamlines became chaotic, with new vortexes found in the lower portion of the previous locations of inferior turbinate and inferior meatus (No. 2-4 and 6) (Figure 7).

**Figure 8:**
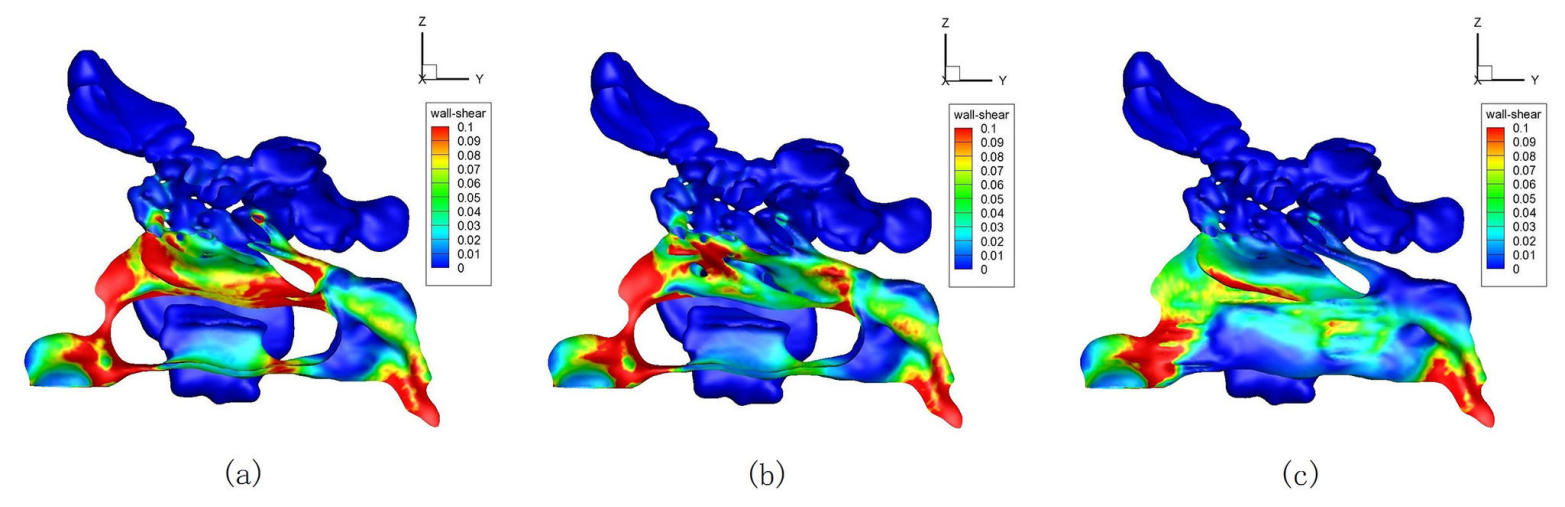
Wall shear stress distributions in the normal (a), ENS-MT (b) and ENS-IT (c) models (No. 1).

4. Changes of the wall shear stress were similar in all the 7 ENS-MT/ENS-IT models, respectively. The structures of the No. 1 model were representatively illustrated in Figure 8. 

In the normal models, wall shear stresses were higher in the nasal valve and head of the inferior turbinate. In the ENS-MT models, wall shear stress distributions changed slightly- decreased in the middle-posterior portions of the (previous) middle turbinates. In the ENS-IT models, wall shear stresses decreased widely, and the areas with larger wall shear stresses shrinked greatly (No. 1, 3-7). 

## Discussion

In our study, the nasal aerodynamic features of the typical ENS-IT and ENS-MT models have been obtained by CFD simulation. The changes in the ENS-IT models are generally consistent with the results in most previous studies[5,6]: decreased nasal resistance, velocities and wall shear stresses, and more chaotic streamlines. In the ENS-MT models, the changing trends of the measured parameters are similar to, while less remarkable than those in the ENS-IT models. Velocities increased significantly around sphenopalatine ganglion in the ENS-MT nasal structures during both inspiration and expiration. To fill the knowledge gap by obtaining the aerodynamic features in the typical ENS-MT nasal structures is one of the major contributions of our study.

As speculated by other researchers[1], the occurrence of ENS can be explained or at least partially explained by the aerodynamic changes in the ENS-IT/ENS-MT models. In the following part, we are going to mainly focus on finding the aerodynamic origins of the development of ENS symptoms, based on the findings in our study.

1. Dryness and crusting. In the ENS-IT models, more chaotic streamlines and new vortexes were shown near the bottom wall of the nasal cavities (Figure 7), where crusting is frequently found in patients with ENS-IT. More water vapors are taken away by the chaotic airflows and vortexes[8-11], thus the feeling of dryness is more likely to generate and the crusting is more easily formed. 

2. Paradoxical nasal obstruction. It is the most characteristic manifestation in ENS-IT patients. Meanwhile it is the most confusing one for otorhinolaryngologists, due to the inconsistency between patients’ complaint of nasal obstruction and the wide-open nasal cavities in the inspection[1,12]. This makes it more meaningful to better understand the corresponding pathogenesis. In our study, the velocities decrease widely in both phases of respiration in the ENS-IT nasal structures, compared to those in the normal noses (Figure 2, 4 and 6). As the velocities of airflows are one of the specific stimulations to mechanoreceptors in nasal mucous- a receptor participating in the nasal airflow sensation[1,3-17], a reduction in velocity would lead to decreased sensation of airflow. It is exactly a frequent description of paradoxical nasal obstruction by patients with ENS-IT[1]. 

On the other hand, higher temperatures on thermoreceptors in the nasal mucous, which participate together with mechanical receptors in the nasal airflow sensation, have been shown to contribute to the subjective nasal obstruction[13,18]. Although we did not obtain the temperature gradients directly in our study, they can be satisfactorily reflected by the wall shear stress distribution- areas with higher shear stresses have lower temperatures, and vice versa[19]. In our study, wall shear stresses decrease broadly in the ENS-IT models (Figure 8), which indicates a wide increase of the nasal temperatures, thus may lead to the subjective nasal obstruction in this group of patients 

1. Hyposmia or anosmia. In ENS-IT models, air fluxes in the olfactory areas significantly decreased (Figure 7, Table 1) (upper middle turbinate, superior turbinate and their corresponding portions on the septum[20-22]). The correspondingly decreased “olfactory particles” transported by the airflows would reduce the stimulation to olfactory sensors, leading to hyposmia, or even anosmia. 

4. Headache in ENS-MT. It is the most typical complaint of patients of this subtype, characterized by its association with respiration[2,12]. Some authors speculated that headache might be caused by the changed or increased stimulation to the sphenopalatine ganglion[1]. Noticeably, in our study, velocities significantly increased around the sphenopalatine ganglion in the ENS-MT models (Figure 3-6, Table 1), thus the stimulation to the ganglion and its accessory nerves by the airflows would increase, which was likely to be the cause of headache associated with respiration. 

The aforementioned common changes of aerodynamic features in ENS-IT were less prominent in ENS-MT, which were possibly responsible for those typical symptoms of ENS (dry nose, crusting, paradoxical nasal obstruction, and hyposmia or anosmia) from the above analyses. That was consistent with the fact that patients with ENS-MT are less likely to develop those symptoms, compared to people with ENS-IT. Similarly, increased velocities around sphenopalatine ganglion in the ENS-MT nasal structures were not found in the ENS-IT ones, which is coherent with that headache is predominantly seen in patients with ENS-MT.

Inter-individual variances of the aerodynamic changes demonstrated in our study would be a reasonable basis of heterogeneity in clinical manifestations among patients with the same subtypes of ENS. For instance, the aerodynamic changes were less remarkable in the ENS-IT model of the No. 2 subject, compared to the other individuals, who might not develop ENS after the radical inferior turbinectomy, or only have mild symptoms in the long-term follow-up. Additionally, velocities around the sphenopalatine ganglion did not increase in all the ENS-MT models, or new vortexes did not appear in all the ENS-IT models. These would explain why some people do not have headache after middle turbinectomy, or others do not suffer that much from a dry nose or crusting after inferior turbinectomy, etc. From the comparison of ENS-IT and ENS-MT, and the analyses of inter-individual variations, we can be more confident about the essential function of nasal aerodynamics in the pathogenesis of ENS.

Though the aerodynamic changes would be able to explain a number of phenomena in ENS, it is difficult to explain the occurrence of ENS-type[13], in which the aerodynamic features change little due to the relatively normal nasal structures. It indicates that there might be other factors responsible for the occurrence of ENS, such as nerve injury with impaired regeneration, etc., where further studies are needed and combined with the aerodynamic studies when possible and necessary.

There are several limitations in our study, as illustrated below. Firstly, the calculations in our study were based on several assumptions- laminar airflow in the nasal cavities and rigid wall, etc., which were not exactly the real situation. Though they were reasonable simplifications based on our prior calculation, and have been accepted and applied by other researchers[5-7] before, it remains worthy of trying to make more accurate estimation by using more complicated models in the future studies, such as the sine-analog one[23] with further refined boundary conditions. Secondly, restricted by the resources available, we did not simulate other nasal structures of ENS, such as the postoperative ones of unilateral radical turbinectomy or partial turbinectomy, etc. Thirdly, although the sample size in our study (n=7) is larger than most published CFD studies (n=1 or 2), it remains small for generating undisputed conclusions on the relationship between the aerodynamic features and clinical manifestations of ENS. The result or hypothesis developed should be further tested in the future studies with larger sample size. Fourthly, the research subjects in our study were all male adults. Nasal aerodynamic changes in female patients need be explored in the future. 

## Conclusions

 Based on 7 different nasal structures, we obtained the aerodynamic features in the two typical nasal structures of ENS-IT and ENS-MT, the latter of which was little known before our study. The changes of nasal aerodynamic features are able to explain a number of typical symptoms of ENS. Less prominent changes of nasal aerodynamic features are consistent with the milder corresponding manifestations. In addition, inter-individual variances in the changes provide a reasonable basis for the heterogeneity of manifestations among patients of the same subtypes. Hence, nasal aerodynamics is very likely to play an essential role in the pathogenesis of ENS. Importantly, a new hypothesis has been proposed that the increased velocities around the sphenopalatine ganglion in the ENS-MT models would be responsible for headache in ENS-MT. However, the results and hypothesis developed need to be validated in further studies with larger sample size and more complicated calculating models in the future. 

## References

[1] Chhabra N, Houser SM. (2009) The diagnosis and management of empty nose syndrome. Otolaryngol Clin North Am 42 (2): 311-330, ix 19328895 10.1016/j.otc.2009.02.001

[2] Payne SC. (2009) Empty nose syndrome: what are we really talking about? Otolaryngol Clin North Am 42 (2): 331-337, ix-x 19328896 10.1016/j.otc.2009.02.002

[3] Scheithauer MO (2010) Surgery of the turbinates and "empty nose" syndrome. GMS Curr Top Otorhinolaryngol Head Neck. Surg 9: Doc03. 10.3205/cto000067 PMC3199827 22073107

[4] Coste A, Dessi P, Serrano E (2012) Empty nose syndrome. Eur Ann Otorhinolaryngol Head Neck Dis 129 (2): 93-97. doi:10.1016/j.anorl.2012.02.001. PubMed: 22513047. 22513047

[5] Chen XB, Leong SC, Lee HP, Chong VF, Wang DY (2010) Aerodynamic effects of inferior turbinate surgery on nasal airflow--a computational fluid dynamics model. Rhinology 48 (4): 394-400. PubMed: 21442074. 21442074 10.4193/Rhino09.196

[6] Wexler D, Segal R, Kimbell J (2005) Aerodynamic effects of inferior turbinate reduction: computational fluid dynamics simulation. Arch Otolaryngol Head Neck Surg 131 (12): 1102-1107. doi:10.1001/archotol.131.12.1102. PubMed: 16365225. 16365225

[7] Na Y, Chung KS, Chung SK, Kim SK (2012) Effects of single-sided inferior turbinectomy on nasal function and airflow characteristics. Respir Physiol Neurobiol 180 (2-3): 289-297. doi:10.1016/j.resp.2011.12.005. PubMed: 22227321. 22227321

[8] Elad D, Wolf M, Keck T (2008) Air-conditioning in the human nasal cavity. Respir Physiol Neurobiol 163(1-3): 121-127. doi:10.1016/j.resp.2008.05.002. PubMed: 18565805. 18565805

[9] Garcia GJ, Bailie N, Martins DA, Kimbell JS. (2007) Atrophic rhinitis: a CFD study of air conditioning in the nasal cavity. J Appl Physiol (1985) 103(3):1082-1092 10.1152/japplphysiol.01118.2006 17569762

[10] Naftali S, Rosenfeld M, Wolf M, Elad D (2005) The air-conditioning capacity of the human nose. Ann Biomed Eng 33(4): 545-553. doi:10.1007/s10439-005-2513-4. PubMed: 15909660. 15909660

[11] Scheithauer MO (2010) Surgery of the turbinates and "empty nose" syndrome. GMS Curr Top Otorhinolaryngol Head Neck. Surg 9: Doc03. 10.3205/cto000067 PMC3199827 22073107

[12] Houser SM (2006) Empty nose syndrome associated with middle turbinate resection. Otolaryngol Head Neck Surg 135 (6): 972-973. doi:10.1016/j.otohns.2005.04.017. PubMed: 17141099. 17141099

[13] Houser SM (2007) Surgical treatment for empty nose syndrome. Arch Otolaryngol Head Neck Surg 133 (9): 858-863. doi:10.1001/archotol.133.9.858. PubMed: 17875850. 17875850

[14] Clarke RW, Jones AS, Charters P, Sherman I (1992) The role of mucosal receptors in the nasal sensation of airflow. Clin Otolaryngol Allied Sci 17 (5): 383-387. doi:10.1111/j.1365-2273.1992.tb01679.x. PubMed: 1458618. 1458618

[15] Wrobel BB, Bien AG, Holbrook EH, Meyer GE, Bratney NA et al. (2006) Decreased nasal mucosal sensitivity in older subjects. Am J Rhinol 20 (3): 364-368. doi:10.2500/ajr.2006.20.2862. PubMed: 16871945. 16871945

[16] Wrobel BB, Leopold DA (2005) Olfactory and sensory attributes of the nose. Otolaryngol Clin North Am 38 (6): 1163-1170. doi:10.1016/j.otc.2005.07.006. PubMed: 16326176. 16326176

[17] Frasnelli J, Heilmann S, Hummel T (2004) Responsiveness of human nasal mucosa to trigeminal stimuli depends on the site of stimulation. Neurosci Lett 362 (1): 65-69. doi:10.1016/j.neulet.2004.02.059. PubMed: 15147782. 15147782

[18] Yogeetha R, Raman R, Quek KF (2007) Effects of temperature changes on nasal patency. Singapore Med J 48 (4): 304-306. PubMed: 17384876. 17384876

[19] Elad D, Naftali S, Rosenfeld M, Wolf M (2006) Physical stresses at the air-wall interface of the human nasal cavity during breathing. J Appl Physiol (1985) 100 (3): 1003-1010. PubMed: 16269523. 16269523 10.1152/japplphysiol.01049.2005

[20] Pinto JM (2011) Olfaction. Proc Am Thorac Soc 8 (1 ): 46-52. doi:10.1513/pats.201005-035RN. PubMed: 21364221. 21364221 PMC3131780

[21] Leopold DA, Hummel T, Schwob JE, Hong SC, Knecht M et al. (2000) Anterior distribution of human olfactory epithelium. Laryngoscope 110 (3 Pt 1): 417-421. PubMed: 10718430. 10718430 10.1097/00005537-200003000-00016

[22] Lane AP, Gomez G, Dankulich T, Wang H, Bolger WE et al. (2002) The superior turbinate as a source of functional human olfactory receptor neurons. Laryngoscope 112 (7 Pt 1): 1183-1189. PubMed: 12169895. 12169895 10.1097/00005537-200207000-00007

[23] Song J (2011) Nasal aerodynamic study on unsteady respiration. Doctoral dissertation, Peking Union Medical College.

